# Detection of Epstein-Barr Virus DNA in Gastric Biopsies of Pediatric Patients with Dyspepsia

**DOI:** 10.3390/pathogens9080623

**Published:** 2020-07-30

**Authors:** Abigail Morales-Sánchez, Javier Torres, María G. Cardenas-Mondragón, Carolina Romo-González, Margarita Camorlinga-Ponce, Lourdes Flores-Luna, Ezequiel M. Fuentes-Pananá

**Affiliations:** 1 Research Unit on Virology and Cancer, Children’s Hospital of Mexico Federico Gomez, Mexico City 06720, Mexico; abmorales@himfg.edu.mx; 2 Infectious Diseases Research Unit, CMNS-XXI, Mexican Institute of Social Security (IMSS), Mexico City 06720, Mexico; jtorresl57@yahoo.com.mx (J.T.); carlupita@yahoo.com.mx (M.G.C.-M.); margaritacamorlinga@yahoo.com (M.C.-P.); 3 Laboratory of Experimental Bacteriology, National Institute of Pediatrics, Mexico City 04530, Mexico; crgaro_06@yahoo.com.mx; 4 Research Center in Population Health, National Institute of Public Health, Cuernavaca 62100, Mexico; mflor@insp.mx

**Keywords:** *Epstein-Barr Virus*, helicobacter pylori, pediatric dyspepsia, gastric inflammation, nonatrophic gastritis

## Abstract

In this study, we assessed the presence of *Epstein-Barr virus* (EBV) in gastric samples derived from pediatric patients with dyspeptic symptoms, aiming to understand whether EBV participates in the development of early gastric lesions influencing chronic inflammation, in conjunction with the *Helicobacter pylori* (Hp) bacterium. We analyzed EBV load in 236 gastric biopsies derived from 186 pediatric patients with chronic dyspepsia and compared it with EBV serology, Hp load and serology, and with immune cell infiltration. We found that 7.5% of patients were positive for EBV load, ranging from 240 to 29,685 genomic copies/μg of DNA. Hp genomic sequences were found in 24.7% of patients. EBV positive samples did not correlate with Hp status and were characterized by absent to moderate immune cell infiltration. To our knowledge, this is the first study addressing EBV load in the stomach in a large cohort of pediatric patients with dyspeptic symptoms, providing evidence of EBV localization in the gastric mucosa in early inflammatory lesions. The lack of correlation between EBV and both Hp infection and inflammation is perhaps explained by independent pathogenic mechanisms or because of the randomness of the gastritis sampling. This is also supported by a moderate association between EBV load and serology.

## 1. Introduction

Gastric cancer (GC) emerges within a context of persistent inflammation, in which a chronic gastritis is the earliest lesion that is already present in some children [1]. Chronic gastric inflammation is associated with persistent *Helicobacter pylori* (Hp) infection, a stomach resident bacterium with a plethora of mechanisms to counteract the acidic pH and colonize the gastric mucosa. About 10% of GC are associated with Epstein-Barr virus (EBV) infection, a persistent pathogen most often acquired in early childhood. EBV is considered a lymphotropic virus because it mainly infects and persists in B lymphocytes. Yet, EBV is also able to infect epithelial cells, which facilitates viral transmission to new hosts [2]. For this, EBV colonizes the Waldeyer’s ring lymph nodes in the oropharynx, where epithelial cells and lymphoid tissue intimately coexist [2]. How and when EBV reaches the gastric mucosa remains unclear, as well as whether it may contribute with dyspeptic symptoms in early gastric lesions.

We have previously reported that elevated levels of antibodies directed against EBV lytic proteins significantly correlate with advanced gastric lesions and with enhanced stomach infiltration of polymorphonuclear (PMN) and mononuclear (MN) immune cells, supporting the idea that EBV reactivation in the stomach is also contributing to the chronic inflammatory process, in conjunction with Hp [3,4,5]. Indeed, we observed that even children younger than ten years old with highly inflammatory and symptomatic gastritis already had elevated levels of anti-EBV antibodies [3]. In this study, we aimed to find evidence of viral loads already present in the gastric mucosa of children with dyspepsia, and address whether or not load correlated with gastric inflammation markers and Hp status.

## 2. Materials and Methods

### 2.1. Patients and Clinical Samples

This study was approved by the Ethical, Biosafety, and Scientific Institutional Review Boards of the Children’s Hospital of Mexico “Federico Gómez” and the Pediatrics Hospital of the Mexican Social Security Institute. The study was performed on frozen archived samples, parents or guardians of the enrolled patients agreed to sign a letter of consent. All patients attended the gastroenterology unit due to dyspeptic symptoms characterized by chronic abdominal pain present for at least six months. Clinical diagnosis as nonatrophic gastritis was based on endoscopy, histology, and clinical presentation. Patients under treatment with antiacid, antibiotics or proton pump inhibitors during the three weeks prior to sample collection were excluded.

Blood and gastric tissue samples were collected at the time of diagnosis. The study started with 356 frozen samples corresponding to 257 patients. Paired samples of the stomach antrum and corpus were collected from some of the patients. One biopsy sample was used for DNA extraction and estimation of viral and bacterial loads by PCR-based approaches. The extracted DNA was first tested for quality using a qPCR directed against the endogenous β-actin gene, only samples with good DNA quality were chosen to continue in the study. Overall, the samples we included in the study were the following—236 gastric tissue samples derived from 186 patients; only antrum tissues from 86 patients, only corpus tissues from 50 patients, and paired antrum and corpus tissues from 50 patients, for a total of 136 samples from antrum and 100 samples from corpus. Of the 186 patients, we had information of EBV and Hp serology for 134 (73%) of them. Serology and viral load results were compared with the histological examinations present in the patient’s clinical records, for which we had information about the Sydney histological evaluation of the inflammatory status for 107/136 (79%) antrum samples, and 84/100 (84%) corpus samples.

### 2.2. EBV Genome Quantification

Total DNA was purified from antrum and corpus tissues using a QIAamp DNA Mini Kit (Qiagen, Hilden, NRW, Germany) according to the manufacturer’s instructions. DNA quantification and purity were evaluated in a nanodrop 2000 spectrophotometer (Thermo Fisher Scientific, Waltham, MA, USA). Integrity and presence of PCR inhibitors in DNA were checked by amplification of the endogenous human β-actin gene. Detection of EBV-Balf5 gene and endogenous human β-actin gene was carried out simultaneously by a TaqMan probe-based qPCR in duplex format. Only samples with early β-actin Ct (up to 24) were included in the study. For quantification of EBV viral load, we constructed a standard curve using 10-fold serial dilutions (10^6^–10^1^ copies) of a plasmid containing a fragment of the EBV-Balf5 gene. Every standard also contained 100 ng of DNA of an EBV negative human cell line as background DNA. The PCR assays were performed in a final volume of 25 μL containing 1x QuantiTect Multiplex PCR NoRox (Qiagen, Hilden, NRW Germany), 250 nM of each primer, 125 nM of EBV-Balf5 probe/62.5 nM of β-actin probe, and 50 or 100 ng of DNA as template (according to the availability of DNA). The EBV probe was labeled with 6-Carboxyfluorescein (FAM) fluorophore, while the β-actin probe was labeled with Cyanine 5 (Cy5) fluorophore. Primers and probes have been previously reported [6] and were commercially synthesized (Synthetic DNA S.A.P.I. of C.V., Gto, Mexico). The cycling conditions were the following—a uracil-DNA glycosylase (UDG) incubation step at 52 °C for 2.5 min, an initial denaturation and polymerase activation step at 95 °C for 15 min, followed by 50 cycles of denaturation at 95 °C for 15 sec and annealing/elongation at 60 °C for 1 min. The limit of detection with 95% confidence of the PCR test was determined at 21 EBV genomic copies [6].

### 2.3. Helicobacter Pylori PCR Determination

The identification of Hp in the gastric biopsy DNA was performed by endpoint PCR using ureC (*glmM*) and 16sRNA targeting primers [7]. The reaction mixture (25 μL) conditions were as follows—1.5 mM magnesium chloride, 0.2 mM dNTPs, 5 U Taq DNA Polymerase (Thermo Scientific Inc, Waltham, MA, USA), and 0.5 mM of each primer. The amplification conditions were 94 °C for 5 min, followed by 35 cycles (92 °C for 30 sec, 55 °C for 40 sec, 72 °C for 40 sec); and a final extension of 72 °C for 5 min. The final amplification products were of 294 (*glmM*) and 522 bp (16sRNA). The reaction products were run on a 2% agarose gel, which was then stained with Midori Green Advance (Nippon Genetics Co, Dueren, Germany). Results were considered positive if one of the PCR products was observed.

### 2.4. Serologic Determination of Anti-EBV VCA and Anti-Hp Antibodies

Antibodies directed against the viral capsid antigen (VCA) were evaluated using the following ELISA kits—quantitative anti-EBV VCA IgG assay (HUMAN, cat, 51204) and qualitative anti-EBV VCA IgM assay (HUMAN, cat. 51104). ELISAs were performed following the manufacturer´s recommendation and as we have previously reported [3,4]. Antibody titers were calculated according to manufacturer´s instructions and expressed as Human Units (HU)/mL. Evaluation of anti-Hp CagA and anti-Hp antibodies was done using in-house ELISA assays as previously described [8,9]. One hundred μL/well of a 1:500 dilution of a sonicated extract (1 mg/mL) of a mixture of three Hp strains isolated from Mexican patients were placed in the ELISA plates, washed, and an equal volume of a 1:400 dilution of sera was placed in the plate, followed by 100 μL/well of a 1:1000 dilution of an antihuman IgG conjugated to alkaline phosphatase (SouthernBiotech, Birmingham, AL, UK). The substrate used was p-nitrophenylphosphate, 1 mg/mL (Southern Biotech, Birmingham, AL, UK). The plates were incubated under dark conditions at 37 °C for 20 min, after which 50 µL of stop solution was added into each well. Absorbance was read at 405 nm. The CagA antigen preparation was kindly supplied by OraVax, Cambridge, MA and consisted of a recombinant peptide at a protein concentration of 8 mg/mL that was used at a 1 μg/well. Samples were considered positive for CagA and Hp when ELISA units were ≥1.5 and ≥1.0, respectively, according to the validated cut-offs in Mexican population [8]. Each plate included four positive and four negative serum samples, all samples were analyzed in duplicate.

### 2.5. Histopatological Examination

Antrum and corpus tissues were formalin-fixed, paraffin-embedded, and stained with hematoxylin-eosin for histologic examination. A single experienced pathologist evaluated the inflammatory status according to the Sydney system. PMN and MN cell infiltration was graded from absent to severe compared with published diagrams (analogue scales).

### 2.6. Statistical Analysis

Stata software package v14.1 (for Windows) was used for performing all data analyses and GraphPad Prism 8.4.0 (for OS X) was used for constructing graphs. A descriptive analysis of the age and sex of pediatric patients was performed. A proportion test was used to evaluate differences of frequencies in the compared groups of Table 1. A nonparametric Mann–Whitney U test was used for comparing two groups of continuous variables—viral load in antrum vs. cardia; EBV-VCA IgG titers in EBV-negative vs. EBV-positive tissues and EBV viral load in EBV-VCA IgG low vs. EBV-VCA IgG high. A nonparametric Kruskal–Wallis test was used to compare EBV viral load in the Hp antibody titer subgroups (Figure 2). Fisher’s test used to explore the relation between degree of inflammation and presence of EBV in gastric tissues. Crude odds ratio was used to evaluate the relationship between titers of Hp antibodies and presence of EBV in gastric tissues. T Spearman correlation test was used to analyze the correlation between viral load and EBV antibody titers. A *p* < 0.05 was considered statistically significant for all tests.

## 3. Results

### 3.1. Screening of EBV DNA in Gastric Biopsies and Comparison with Systemic Anti-EBV Antibodies

In this study, we aimed to understand whether EBV was already present in the gastric mucosa of children with dyspepsia contributing with gastric inflammation in conjunction with Hp. The study included 186 pediatric patients with dyspepsia characterized by recurrent abdominal pain for at least six months (Table 1), 39.4% male and 60.6% female. After histological examination, the patients were diagnosed with nonatrophic gastritis. We detected 18 (7.6%) EBV positive samples coming from 14 (7.5%) patients. Because the stomach site from which the biopsy is taken is chosen at random, we also analyzed the positivity to Hp, to have a reference of the likelihood to find the pathogen that is the normal resident of the gastric mucosa. Hp was found positive in 50 (22.8% of the samples), almost three times more than EBV (Table 1).

**Figure 1:**
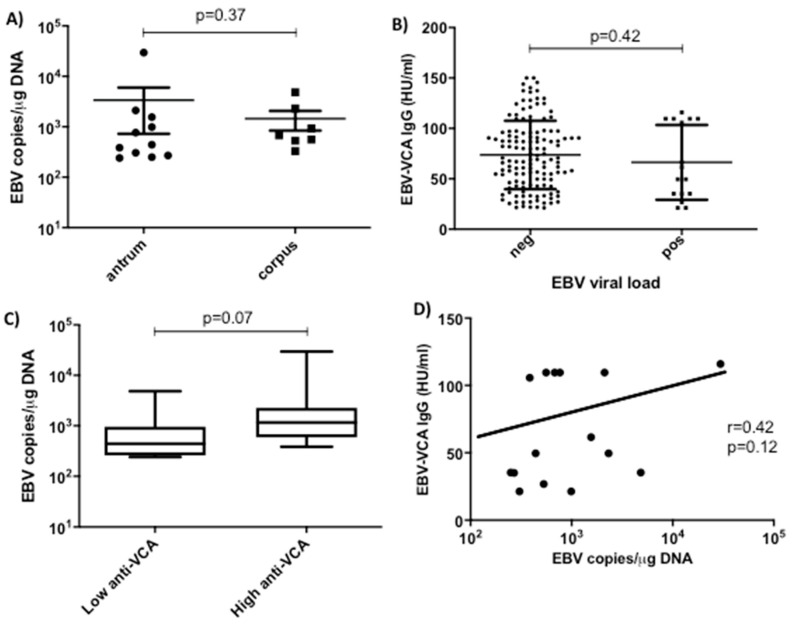
EBV load and (**A**) comparison between antrum and corpus. Shown are the median and the interquartile ranges (Mann–Whitney U test). (**B**) Anti-EBV VCA IgG antibody titers of children with negative (neg) versus positive (pos) viral load in gastric mucosa (Mann–Whitney U test). (**C**) EBV viral load in children with low and high anti-EBV VCA IgG antibody titers. (**D**) Spearman correlation between EBV viral load in gastric mucosa and anti-EBV VCA IgG antibody titers.

EBV-positive samples were found in antrum (*n* = 11) and corpus (*n* = 7), with only four patients with positive samples in both sites. We found no significant differences in either EBV frequency (Table 1) or load (Figure 1A) between antrum versus corpus samples. While only 7.5% of patients were positive to anti-IgM antibodies, 69.8% were positive to anti-IgG VCA antibodies. No patient was anti-IgM positive and anti-IgG negative, indicating that there were no patients in the cohort who had recently been infected with EBV and most likely all had a chronic infection. Elevated anti-IgG VCA often marks recurrent events of reactivation, and we have previously shown in this cohort that elevated anti-IgG VCA is a good marker for severe gastritis [3]. We assessed whether there was a correlation between the gastric biopsy positivity and the systemic anti-EBV antibodies by comparing the anti-IgG antibody levels in patients showing positive and negative viral load. We found no differences between them (Figure 1B). We divided the anti-IgG data into high and low categories using the median as the cut off value. When we compared the viral load in both IgG groups, we found that EBV viral load tended to be higher in the high anti-IgG group (*p* = 0.07, Figure 1C). Indeed, only a moderate correlation was found between serology and viral copy number (*r* = 0.42, *p* = 0.12) (Figure 1D).

### 3.2. Evaluation of Immune Cell Infiltration in EBV Positive Biopsies

MN and PMN cell infiltration were graded from absent to severe in both corpus and antrum. We have also previously observed a correlation between the most severe infiltration and elevated levels of anti-VCA serology [3]. Here, we wanted to assess whether samples that were positive to EBV load were also more infiltrated. Unfortunately, we could only gather data about immune cell infiltration for ten of the samples with EBV positive load. We observed that both antrum and corpus EBV-positive samples exhibited mild to moderate MN cell infiltration, while for PMN cell infiltration, most samples were in the absent group, with only two in the mild, and one in the moderate (Table 2). Statistical analysis showed no associations.

### 3.3. Hp Antibody Evaluation in Patients with EBV-Positive vs. Negative Gastric Tissues

**Figure 2:**
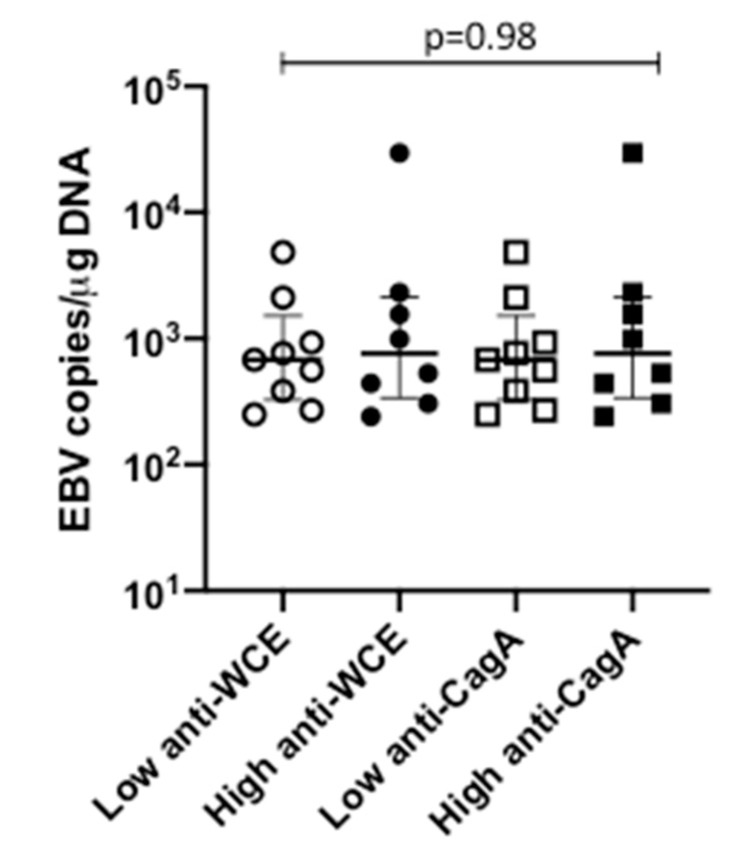
EBV relation with Hp serology. EBV viral load in children with low and high anti-HP whole cell extract (WCE) and anti Hp CagA antibody titers (Kruskal–Wallis test).

Since Hp is the natural inhabitant of the gastric mucosa and EBV resides in B lymphocytes, to look for evidence that Hp infection is part of the mechanism that attracts EBV to the stomach, we wanted to assess whether we could find a correlation between EBV load and the anti-Hp serology. For this analysis, we divided anti-Hp antibody levels (against the whole bacterial extract or against CagA) into two subgroups of high and low antibodies, also using the median as the cut-off value. We observed that EBV-positive samples were distributed almost evenly between both groups of high and low antibody titers (Figure 2 and Table 3). To put these data in context we performed a similar analysis for Hp PCR positivity and Hp serology. Since the estimation of Hp load was not quantitative, we compared positive and negative load vs. positive and negative serology. We found an OR = 3.49 (CI = 1.46–8.56, *p* = 0.0018) and OR = 3.56 (CI = 1.51–8.54, *p* = 0.0011) of being positive to PCR in patients that were serology positive to the Hp whole cell extract or to CagA, respectively. We also could not observe an association between EBV and Hp gastric loads.

Table 4 shows a summary of the data on EBV and Hp status, in which it can be observed that 7/14 (50%) of the EBV PCR positive pediatric patients are negative to Hp infection assessed by either PCR or serology. This data argues that EBV gastric load is independent of Hp infection.

## 4. Discussion

EBV infects close to 95% of the adult population worldwide, with memory B cells being the main reservoir of viral latent infection. There is experimental and clinical evidence of the capacity of EBV to also infect epithelial cells, but while B cells are very efficiently infected and immortalized in vitro, similar infection assays on epithelial cells only result in less than 1% infection, and the viral genome tends to be lost upon serial passages [10,11,12]. Still, EBV has been consistently associated with epithelial cancers. Although today, it is not clear when EBV arrives in the gastric mucosa, and whether it participates in the development of early gastric lesions and in the chronic inflammation that progressively leads to GC.

Our group has formed a cohort of pediatric patients with chronic dyspepsia that attended the Gastroenterology unit of our institution from September 1994 to October 2001 [13,14]. We have previously documented in this cohort that high levels of systemic anti-EBV antibodies, along with high levels of anti-Hp antibodies, correlate with increased infiltration of immune cells [3]. In agreement, we have found that elevated anti-VCA antibodies also mark adults with severe preneoplastic lesions [4]. Here, we detected EBV load in 14 (7.5%) patients oscillating from 240 to 29,685 copies/μg of tissue DNA. Although some studies support that the EBV-associated GC mainly develops in nonantral regions of the stomach [15,16,17], we have previously observed a similar distribution in the stomach corpus and antrum [18]. Here, we also observed EBV in both regions of the stomach. To the best of our knowledge, this is the first study addressing the EBV load in the stomach in a cohort of pediatric patients with dyspeptic symptoms and early gastric lesions.

To put the frequency of EBV-positive patients in context, we also performed an end-point PCR to identify Hp. Evidence of Hp genomic sequences was found in 46 patients (24.7%). Some studies have looked into Hp load by PCR, finding 25–46% and 17–26% positivity in adult and pediatric dyspeptic patients, respectively [19,20,21,22,23,24,25]. PCR detection of Hp infection has not been previously reported for this cohort of patients. Multiple tests are used to evaluate Hp infection, some of them noninvasive and indirect, such as the urease breath test, serology, and PCR of feces. Because Hp mainly inhabits the stomach, these tests are informative of Hp gastric colonization. This is not true for EBV, and when high antibody levels are detected, indicating EBV exacerbated infection, it is not clear what tissue(s) are harboring the virus.

We did not observe an association between EBV load and Hp status, in either load or serology, that supported a mechanism of Hp infection attracting EBV infected B cells to the gastric mucosa. Molecular studies support an intimate interaction between both microorganisms cooperatively enhancing their pathogenic processes [26,27,28,29], although most of those studies are based on in vitro tests and/or in adult patients. Alternatively, both microorganisms may have autonomous mechanisms of pathogenesis in the gastric mucosa, and their presence independently adds to the local tissue damage. Since the association between EBV load and serology was also moderate, these results may also be explained by the randomness of the gastric sample. When taking tumor samples, it is easier to choose the areas that better represent the lesion, while that task is significantly more complex for superficial widespread lesions. Alternatively, it is possible that the extent of EBV replication in B cells within inflamed gastric mucosa is not solely responsible for the elevated anti-VCA IgG responses but rather, relates to a general systemic increase in EBV replication. In this scenario, the detected EBV load may be originating from B cells undergoing lytic replication, which still argues for a potential contribution of EBV in the gastric inflammatory process. Altogether, this and our previous data argue that EBV anti-VCA antibodies are better markers of gastric inflammation than the tissue viral load [3].

Elevated anti-VCA antibodies mark individuals with higher risk to develop nasopharyngeal carcinoma [30,31,32], supporting a mechanism of enhanced EBV reactivation from latently infected B cells favoring enhanced infection of the nasopharyngeal epithelial cells. Others have also made a similar observation for EBV and GC [4,33,34]. We were expecting to find a correlation between EBV load and the severity of immune cell infiltration in the stomach, but most positive samples presented an infiltration ranging from mild to moderate and from absent to moderate for MN and PMN cells, respectively. Unfortunately, we were only able to find data for immune cell infiltration in about 80% of the gastric samples, of which only 10/18 were positive to EBV. Of note, Hp infection was also found to correlate with mild to moderate MN infiltration and absent to mild PMN infiltration in this cohort of patients [13,35].

An association between EBV and pediatric gastritis has been previously documented in clinical case reports. For instance, EBV positivity has been reported in two young patients with dyspepsia [36], and also in 16 yo and 4 yo patients with infectious mononucleosis and gastrointestinal symptoms [37,38]. These studies are particularly important because they have shown positivity to EBV by in situ hybridization (EBER-ISH), and therefore demonstrated EBV infection in epithelial cells. Because in this study we wanted to test for EBV and Hp load and we only had one available biopsy, we were unable to carry out an EBER-ISH to also assess the cell lineage harboring EBV. There is also a case report in which gastritis to cancer progression was observed soon after a nonmyeloablative hematopoietic stem cell transplantation in an Hp negative patient [39], supporting a gastric cancer-triggering role for EBV starting from early inflammatory lesions. Studies addressing the EBV load, EBV cellular localization and expression of latent and lytic genes will be very informative about the viral contribution to early gastric inflammatory lesions.

## 5. Study Limitations

Although the present study shows increased EBV load in gastric lesions of children with dyspepsia, some in the absence of apparent Hp infection, we could not address how EBV is linked to the pathology of the lesion. The study does not discern whether EBV is contributing to the lesion or it is being attracted by presently unknown factors, and its presence is merely passenger/transient. Future studies should address the nature of the EBV-infected cells, whether they are B cells or epithelial cells, and whether there is evidence of EBV reactivation that may be promoting local inflammation.

## Figures and Tables

**Table 1:** Demographic and clinical characteristics of pediatric patients (*n* = 186).

Age (Years) Median (IQR)			10 (6–13)	
Sex N (%)			Male 93 (39.4%) Female 143 (60.6%) ^1^	
		**Positive Samples** **N (%)**	*p* Value ***	**Genome Copies/μg DNA** **Median (IQR)**
**EBV viral load** in stomach *	Total	18 (7.6)		545 (311–975)
	Antrum	11 (4.7)		412.5 (265–1131)
	Corpus	7 (2.9)	0.85	620 (480–1279)
**Hp PCR** **Positivity**	Total	50 (22.8)		ND
	Antrum	34 (15.5)		ND
	Corpus	16 (7.3)	0.42	ND
		**Positive samples** **N (%)**		**Antibody titers** **Median (IQR)**
**EBV** Serology **	VCA IgG	95 (69.8)		67 (42.8–95.2)
	VCA IgM	10 (7.4)	<0.01	ND
**Hp** Serology **	WCE IgG	71 (52.2)		1.53 (1.06–2.5)
	CagA IgG	44 (32.3)	*p* = 0.03	3.76 (2.1–7.6)

EBV viral load was evaluated in 236 tissue samples (136 of antrum and 100 of corpus) coming from 186 patients. ** EBV and HP serology was evaluated for 136 patients. *** Test of proportions. EBV: Epstein-Barr Virus, Hp: *Helicobacter pylori*, VCA: viral capsid antigen, CagA: cytotoxin-associated gene A, WCE: Whole-cell extract, IQR: Interquartile range, ND = Not determined. ^1^ EBV viral load was evaluated in 236 tissue samples (136 of antrum and 100 of corpus) coming from 186 patients.

**Table 2:** Degree of infiltration of mononuclear and polymorphonuclear cells in the gastric mucosa of children.

Gastric Tissues		Antrum *n* = 107			Corpus *n* = 84		
EBV Viral Load Status in Gastric Mucosa		EBV Neg *n* = 100	EBV Pos *n* = 7	*p* Value **	EBV Neg *n* = 81	EBV Pos *n* = 3	*p* Value **
Mononuclear cell Infiltration * N (%)	Absent	6 (6)	0 (0)		2 (2)	0 (0)	
	Mild	69 (69)	4 (57)		71 (88)	3 (100)	
	Moderate	17 (17)	3 (43)		8 (10)	0 (0)	
	Severe	8 (8)	0(0)	0.42	0 (0)	0 (0)	1.00
Polymorphonuclear cell Infiltration * N (%)	Absent	71 (71)	4 (57)		67 (83)	3 (100)	
	Mild	15 (15)	2 (29)		12 (15)	0 (0)	
	Moderate	9 (9)	1 (14)		1 (1)	0 (0)	
	Severe	5 (5)	0 (0)	0.51	1 (1)	0 (0)	1.00

Mononuclear and polymorphonuclear cell infiltration data were available for 107/136 (79%) samples of antrum, and for 84/100 (84%) samples of corpus. ** Fisher’s test.

**Table 3:** EBV correlation with Hp serology. EBV viral load in children with low and high anti-HP whole cell extract (WCE) and anti Hp CagA antibody titers.

	Anti-Hp Whole Extract (N)			
		Low	High	OR * (95% CI)
EBV viral load (N)	Neg	87	92	1.6 (0.15–1.60) *p* = 0.20
	Pos	11	6	
	**Anti-CagA (N)**			
EBV viral load (N)	Neg	88	91	0.68 (0.21–2.07) *p* = 0.45
	Pos	10	7	

* Crude Odds Ratios.

**Table 4:** Summary of data of EBV PCR-positive patients.

Biopsy	EBV Copies/μg DNA	EBV-VCA IgG (HU/mL)	Hp PCR	Hp-WCE IgG	Hp-CagA IgG
1	240	neg	pos	neg	neg
2 ^a^	250	35.26	neg	neg	neg
3	270	35	neg	neg	neg
4	305	21.26	pos	1.2607	3.7933
5	330	unknown	neg	neg	neg
6	385	105.67	neg	neg	neg
7 ^b^	440	49.59	neg	1.9366	neg
8	530	26.8	neg	neg	1.816
9 ^c^	560	109.58	neg	neg	neg
10 ^d^	680	109.55	neg	neg	neg
11 ^c^	765	109.58	neg	neg	neg
12	930	neg	neg	neg	neg
13	990	21.31	neg	1.086	neg
14	1555	61.61	pos	1.3151	2.126
15 ^d^	2115	109.55	neg	neg	neg
16 ^b^	2325	49.59	neg	1.9366	neg
17 ^a^	4830	35.26	neg	neg	neg
18	29685	115.9	pos	2.2	2.935

Superscript letters indicate two samples from the same patient, four patients were EBV PCR-positive in the antrum and corpus (a–d). Shadowed rows indicate EBV PCR-positive patients that were negative to H. pylori (Hp) status.
